# Solution-focused brief therapy combined with mindfulness-based stress reduction to alleviate claustrophobia during MR imaging

**DOI:** 10.1097/MD.0000000000043190

**Published:** 2025-07-11

**Authors:** Liyuan Fu, Minghui Mao, Chengkun Hong, Yuhang Zhang, Chujie Chen, Zhijie Yang, Yamei Lin, Hui Xiao, Hao Huang

**Affiliations:** a Fuzong Teaching Hospital of Fujian University of Traditional Chinese Medicine (900th Hospital), Fuzhou, Fujian Province, China; b Department of Radiology, 900th Hospital of PLA Joint Logistic Support Force, Fuzhou, Fujian Province, China; c College of Integrative Medicine, Fujian University of Traditional Chinese Medicine, Fuzhou, Fujian Province, China; d Academy of Integrative Medicine, Fujian University of Traditional Chinese Medicine, Fuzhou, Fujian Province, China; e Medical Image Center, The 95th Hospital of Putian in China RongTong Medical Health Corporation, Putian, Fujian Province, China.

**Keywords:** anxiety, magnetic resonance imaging, mindfulness-based stress reduction, phobic disorders, solution-focused approach

## Abstract

**Background:**

This study evaluates the influence of solution-focused approaches combined with mindfulness-based stress reduction (MBSR) on claustrophobia in a high-risk cohort undergoing magnetic resonance (MR) imaging.

**Methods:**

In the prospective observational 2-group study, a total of 77 patients (55% male, mean age 50.1 ± 11.9) underwent solution-focused brief therapy (SFBT) and MBSR before MR examination. The control group (146 patients, 48% male, mean age 48.7 ± 12.9) received the same MR examination condition and only received routine psychological intervention. Before the intervention, after MR examination, all patients were required to complete the Claustrophobia questionnaire, hospital anxiety and depression scale, and the Connor-Davidson resilience scale questionnaire. Additionally, the occurrence of claustrophobic events, image quality, patient satisfaction, average completion time, and 1-time pass rate were recorded. Claustrophobia events included premature termination, sedation, and non-sedation coping actions. The Shapiro–Wilk test was employed to assess the normality of the data, while either an independent samples *t* test or Mann–Whitney *U* test was utilized to analyze differences in quantitative information between the 2 groups. *χ*^2^ test was employed to validate the qualitative data.

**Results:**

The prevalence of claustrophobia was significantly reduced in the intervention group compared with the control group (18%, 14/77 vs 46%, 67/146; *P* < .05), while the need for sedation (3%, 2/77 vs 10%, 15/146; *P* < .05) and non-sedation coping actions (12%, 9/77 vs 23%, 33/146; *P* < .05) was also significantly reduced in patients who received SFBT & MBSR interventions. Patients who received the SFBT & MBSR intervention had lower mean examination completion time (17.28 ± 3.36 vs 19.75 ± 4.23; *P* < .05), and hospital anxiety and depression scale score (from 9.03 ± 0.9 to 6.30 ± 0.8; 6.30 ± 0.8 vs 9.08 ± 1.0; *P* < .05), higher 1-time pass rate (82% vs 61%; *P* < .05), image quality (*P* < .05), examination satisfaction (*P* < .05), and Connor-Davidson resilience scale score (from 57.73 ± 4.5 to 66.08 ± 4.7; 66.08 ± 4.7 vs 57.78 ± 4.6; *P* < .05). However, there was no statistically significant change observed in the Claustrophobia questionnaire for either the intervention or control group.

**Conclusion:**

The intervention model combining SFBT with MBSR may substantially reduce the incidence of claustrophobia in high-risk patients during MR examinations and may reduce the need for sedation and non-sedation coping actions.

## 1. Introduction

Magnetic resonance imaging (MRI) is one of the commonly used clinical imaging methods, which can be used for the diagnosis of a variety of organ diseases without any damage to the human body.^[1]^ Although MRI has the advantages of being noninvasive, non-radioactive, and safe, etc. Compared to other imaging tests (X-ray, CT, ultrasound, etc), it is time-consuming, noisy, and confined. MRI is the most common examination of the abdomen, which requires the patient to breathe evenly and hold his/her breath during the examination.^[2]^ Thus, patient collaboration plays a crucial role in the quality of MRI. However, due to severe claustrophobia caused by the small diameter of the MRI tube or loud noises, up to 10% of patients are unable to undergo MR examinations.^[3,4]^ Previous studies have shown that patients with pacemakers and implantable cardioverter-defibrillators can undergo MR examinations with proper precautions. Thus, severe claustrophobia is currently the main relative contraindication to MRI.^[5,6]^ Patients with claustrophobia may experience various degrees of claustrophobia during the MR examinations due to the confined and noisy environment and the long duration of the examination.^[7]^ Patients with mild symptoms may affect their physical and mental comfort, while those with severe symptoms may not be able to complete the MR examinations successfully and may even suffer from cardiac arrest, which seriously affects the patient’s physical and mental health.^[8]^ Therefore, it is necessary to provide effective medical interventions for claustrophobic patients to alleviate their psychological discomfort and ensure that the MR examinations can be performed smoothly.

The use of open MRI devices or mild sedation is commonly employed to alleviate the adverse effects of claustrophobia in patients requiring MRI scans.^[4,9,10]^ However, the universal substitution of conventional MRI devices for open MRI devices is not feasible, as their utilization is limited due to higher costs and restricted availability in smaller healthcare facilities.^[11]^ The use of sedation, although cost-effective and widely accessible in comparison to open MRI devices, typically necessitates the patient’s conscious participation during the examination process and may potentially lead to various complications.^[12,13]^ To optimize the healthcare experience for patients with claustrophobia across all hospital settings and mitigate medication-related risks, alternative therapeutic approaches have been implemented to alleviate psychological distress during MR examinations^.[11,14]^ The solution-focused brief therapy (SFBT) has been clinically demonstrated to effectively alleviate patient distress and minimize the reliance on medication during medical procedures.^[15]^ This model has been proven effective in enhancing the psychological well-being of individuals with drug addiction and cancer, which can continuously promote the active participation of individuals to achieve the effect of strengthening their problem-solving ability.^[16]^ Mindfulness-based stress reduction (MBSR) is a psychotherapy based on “mindfulness” and meditation on emotions and stress.^[17]^ Numerous studies have demonstrated the efficacy of MBSR in guiding patients to alleviate symptoms of stress, anxiety, and depression through meditation practices, positive breathing, body scanning, and positive meditation to achieve the effect of reducing their negative emotions.^[18–20]^ Although these 2 methods have been clinically proven to be effective in alleviating negative emotions in patients during treatment, there have been no reports of their application to patients with MR examinations.

We hypothesized that the risk of claustrophobia in patients would increase during the MR examinations. SFBT and MBSR interventions for patients before MR examinations can significantly reduce the risk caused by claustrophobia compared with routine psychological care. The aim of our study was to assess the effectiveness of a combined intervention of SFBT and MBSR in patients at an increased risk of claustrophobia. These patients were compared with a control group who were also at an increased risk but received routine psychological care while being examined on the same MRI scanner. The present study focused on the occurrence of claustrophobic events, the patient’s satisfaction with the combined intervention of SFBT & MBSR, the quality of images, the completion of the MR examination, and the results of their various questionnaires.

## 2. Methods

### 2.1. Study design

This study complied with the Declaration of Helsinki and was approved by the Ethics Committee of the 900th Hospital of Joint Logistics Support Force under the approval number 2022-098. A total of 223 consecutive adult patients were referred on weekdays from 7:30 am until 5:30 pm between May 1, 2023, and January 1, 2024. All patients in both the intervention and control groups gave written informed consent. All patients completed a Chinese-translated version of the Claustrophobia questionnaire (CLQ) before and after the examination.^[21]^ The CLQ consists of 26 questions, each of which describes the fear that the patient may experience in different situations and is scored according to the degree of fear from 0 (no fear) to 4 (maximum fear).^[21]^ The average score is calculated by dividing the total sum of scores for all questions by the number of questions. Only questionnaires with all 26 questions answered were considered valid. The specific events and reasons that resulted in the premature termination of the MR examination were meticulously documented (e.g., Claustrophobia, low-quality MRI images due to motion artifacts, pain, etc). For patients who develop severe claustrophobia during the examination, we will immediately terminate the procedure and administer benzodiazepines intravenously or orally if necessary. Non-sedation coping actions included a pause, an escort during the examination, and a specific comfort talk for patients with claustrophobic episodes.

#### 2.1.1. Inclusion criteria

(1) Meets the diagnostic criteria for claustrophobia^[22]^: fear doesn’t match the situation; inability to eliminate bad feelings with explanations or reasoning; inability to self-control bad moods; evasive responses to feared environments.
(2) Voluntary participation in this project.
(3) To undergo MR examinations of any part of the body.

#### 2.1.2. Exclusion criteria

(1) Patients referred from the intensive care unit.
(2) Serious emergency patients.
(3) poor general health status.
(4) Poor psychological condition that prevents cooperation with the examination.
(5) Invasive procedures are required during MR examinations. (e.g., biopsies).
(6) Age <18.
(7) Inability to communicate effectively verbally and illiteracy.
(8) Insufficient time for examinations.
(9) Part of the regular routine but planned for other studies.
(10) Refused participation.

**Figure 1.**
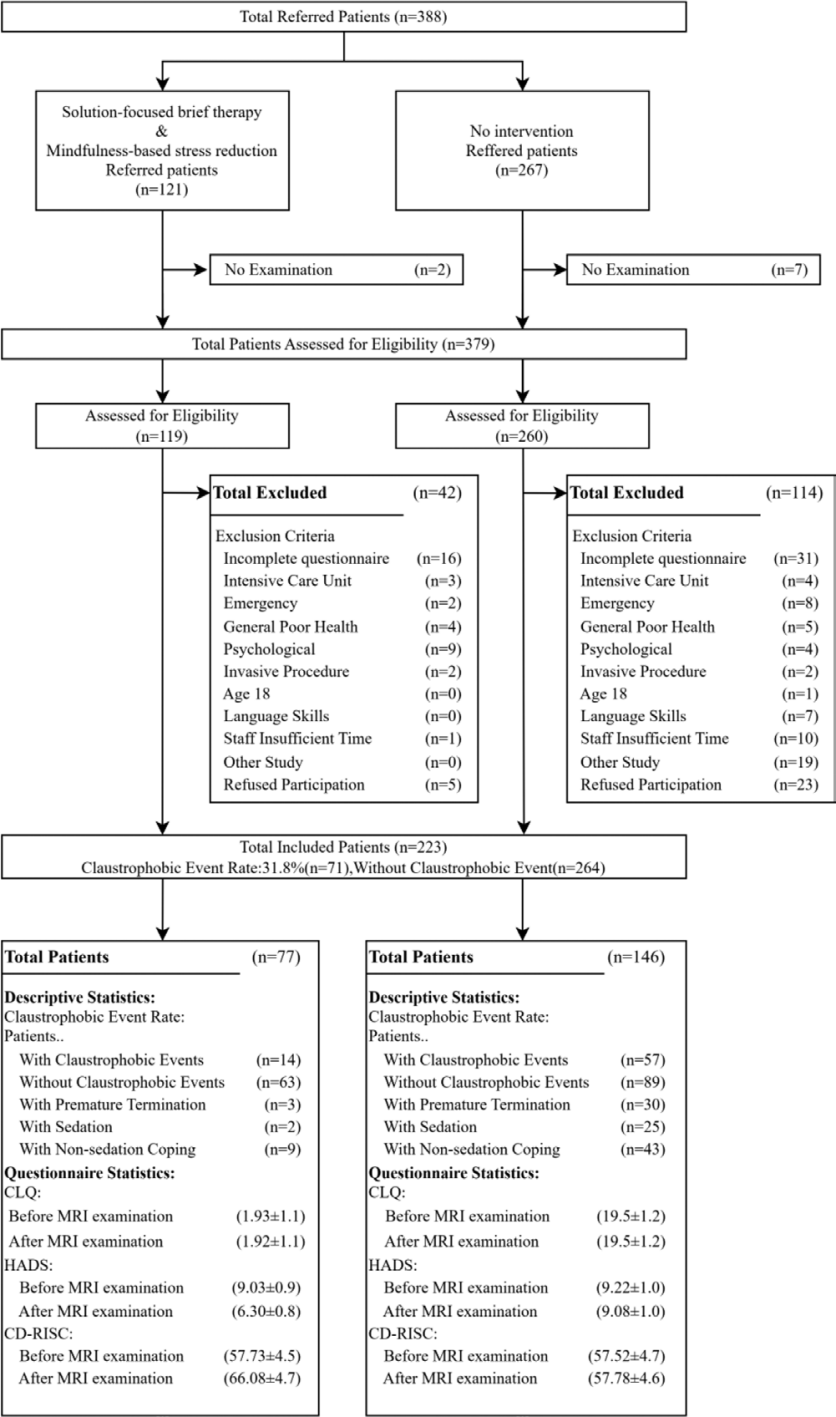
Flow chart of study inclusion in the intervention (solution-focused brief therapy & Mindfulness-based stress reduction) and control groups (routine psychological care). CLQ = Claustrophobia questionnaire, HADS = hospital anxiety and depression scale, CD-RISC = Connor-Davidson resilience scale.

During the study period, patients in the 2 cohorts (1 intervention group and 1 control group) were guaranteed to be the same except for the different modes of intervention (Fig. 1). At the end of the examination, patients were asked to complete the hospital anxiety and depression scale (HADS) and the Connor-Davidson resilience scale (CD-RISC) in addition to the CLQ for cross-sectional comparisons between groups. The HADS is a screening scale developed by Zigmond and Snaith in 1983 and applied primarily to anxiety and depressive symptoms in patients.^[23]^ This scale consists of 14 items, 7 of which rate depression and 7 rate anxiety. The 2 subscales of depression and anxiety are categorized as 0 to 7 for asymptomatic, 8 to 10 for symptomatic suspicion, and 11 to 21 for symptomatic. The CD-RISC is a scientific scale developed by Connor et al in 2003 to measure the psychological resilience of patients.^[24]^ This scale consists of 25 entries in 3 dimensions: resilience and control, strength and optimism, and is scored on a 5-point Likert scale. Higher scores indicate a higher level of psychological resilience. In addition to counting HADS, CD-RISC, and CLQ scores, we also counted the occurrence of claustrophobia, claustrophobic events in relation to the anatomical location of the examination, the average time to complete the examination, the 1-time pass rate, the quality of the images, and the examination satisfaction. At the same time, in order to minimize the bias caused by subjective scoring, we adopted the following control measures: image quality is independently interpreted by 2 experienced imaging physicians according to uniform evaluation criteria, and consensus is reached through negotiation in case of disagreement; Questionnaires were completed immediately after the examination to reduce recall bias, and only fully answered questionnaires were included for analysis. After all questionnaires were completed, cross-sectional and longitudinal comparisons were made between the 2 groups to fully evaluate the intervention outcomes.

### 2.2. Control group

Patients in the control group were treated with routine psychological care:

1. Explain the principle of MR examination and the importance of MRI for further diagnosis and treatment to the patients, so that they can have sufficient psychological preparation.
2. Create a quiet, comfortable, and clean examination environment.
3. Distract the patient during the examination by watching other examiners’ examinations. The patient’s ears can also be plugged with cotton balls to reduce noise or to play soothing and relaxing music in order to alleviate their tension. Patients in the intervention group were intervened with a combination of SFBT & MBSR.

### 2.3. MBSR intervention

We first treated the patient with the MBSR intervention prior to the MR examinations based on the patient’s consent. This intervention therapy is delivered in the form of courses. All courses are taught by doctors who have been specially trained in MBSR therapy. Prior to instruction, all patients in this intervention would receive a one-on-one orientation interview with a physician, and their care was arranged accordingly. The entire MBSR intervention program consisted of 6 sessions, all of which were delivered in small groups over 6 consecutive weeks. The intervention took place in the radiology department lounge of the 900th Hospital of Joint Logistics Support Force, and all courses were delivered via PowerPoint and on-site training. Table 1 demonstrates the specific measures of this intervention.

**Table 1:** Specific components of the mindfulness-based stress reduction intervention.

Time	Course topic	Details
1st week	Beginning	1. Warm-up phase: Distribute the mindfulness-based stress reduction guidebook to patients, present the mindfulness-based stress reduction team members, explain the course’s goal, arrangements, and precautions, and gain their participation. 2. Intervention phase: Play gentle background music, provide a detailed explanation of the raisin exercise, and offer guidance to patients during its execution. First, patients are instructed to tactually perceive the texture and color of the initial raisin, while consciously observing its entire passage from the oral cavity to the stomach. They are encouraged to acknowledge their intention to swallow when ready for ingestion, repeating this process with subsequent raisins. Additionally, patients are provided with a comprehensive explanation regarding the benefits of MRI and how it would aid them post-examination. Furthermore, they are guided in comprehending and managing potential challenges and stressors, empowering them to confront these pressures by cultivating positive thoughts. 3. Discussion phase: Engage in discussions with patients regarding their emotional state and provide instructional audio materials. In the initial week, 2 10-minute sessions were chosen to facilitate patients’ acquisition of mindful eating skills through online coaching.
2nd week	Mindful breathing	1. Warm-up phase: The patients are provided with an explanation of the fundamental principles of mindful breathing and given explicit instructions on how to perform the breathing technique. 2. Intervention phase: The patients are instructed to assume a comfortable position, close their eyes, and mindfully connect with their emotions, emotional state, and intuition in a serene environment. 3. Discussion phase: Encourage patients to engage in open communication, acknowledge and commend those who demonstrate commitment to their practice, and provide guidance for overcoming any challenges encountered during the process.
3rd week	Body scanning	1. Warm-up phase: Reveal the technique of body scanning to patients and provide them with guidance on its implementation. 2. Intervention phase: The patients are instructed to recline on their backs in a quiet environment, ensuring complete relaxation of their entire body. They are guided to gently close their eyes and consciously redirect their focus towards different areas of the body, attentively observing the sensations in the specific region being prepared for MR examination. 3. Discussion phase: Guide patients to refrain from engaging in self-critical behaviors and encourage patients to openly express their emotions during the scanning process.
4th week	Mindful meditation	1. Warm-up phase: Reveal the fundamental aspects of mindful meditation and provide patients with step-by-step instructions for practicing the exercises. 2. Intervention phase: Instruct patients to assume a cross-legged position in a serene environment, placing their hands gently on their knees, allowing their bodies to relax and engaging in slow and deep breathing techniques. Guide patients to direct their attention towards thoughts and ideas, observing them as they arise, evolve, and eventually dissipate. Subsequently, redirect their focus towards the surrounding environment while remaining aware of any bodily sensations. Direct patients to acknowledge and explore their emotions regarding the upcoming MRI examination without judgment or resistance. 3. Discussion phase: Reassure patients to openly discuss their concerns and negative emotions prior to the examination, as well as potential lifestyle adjustments thereafter. Additionally, assist patients in accurately perceiving the examination and alleviating associated negative emotions.
5th week	Mindful walking	1. Warm-up phase: Provide an explanation of mindful walking and lead patients through corresponding exercises. 2. Intervention phase: Instruct patients to ambulate along an undisturbed corridor, maintaining a parallel and shoulder-width stance with relaxed arms hanging by their sides. Guide patients in recognizing their body’s upright position and cultivating the inclination to move forward. Lastly, direct their attention towards mindful breathing while focusing on the upcoming foot lift, perceiving the alternating forces of gravity, and concluding the process with heightened bodily awareness. 3. Discussion phase: Reinforce patients’ awareness of their bodily sensations while walking mindfully, guiding them to attentively observe and enhance their sensitivity towards the changes occurring in their bodies.
6th week	Bringing mindfulness into daily training	1. Warm-up phase: Provide comprehensive explanations of daily mindfulness-based stress reduction techniques and offer guidance to patients for practicing these methods at home. 2. Intervention phase: The patients are instructed to focus on their breathing upon waking up through WeChat, guiding them to concentrate on a minimum of 5 consecutive breath their attention towards daily activities after awakening, such as brushing teeth, washing face, eating, working, etc. Prior to bedtime, they will receive instructions to refocus their complete at least 5 full breaths. 3. Discussion phase: The session concludes with guiding patients to review the lessons, conduct a thorough examination of the MRI, and actively cultivate the ability to face the present moment with complete awareness while integrating mindfulness-based stress reduction into their daily lives.

MRI = magnetic resonance imaging.

### 2.4. SFBT intervention

On the basis of the patient’s consent, the SFBT intervention was conducted by the nurse in the MRI lounge twenty minutes before the examination. The specific process is as follows:

1. Describing the problem: Communicate warmly and positively with the patients to gain insights into their medical condition, lifestyle choices, and psychological well-being. Encourage patients to openly communicate their inner thoughts and concerns regarding the MR examination, and tailor personalized psychological interventions based on their specific circumstances.
2. Developing well-formed goals: The goals of the MR examination are tailored to the patient’s specific condition and their real-time psychological state.
3. Exploring for exceptions: Reassure patients and provide pre-examination breath-holding training to ensure their positive and optimistic attitude towards the examination. Monitor patients closely during the procedure, promptly addressing any discomfort they may experience and providing corresponding guidance.
4. End of session feedback: Nurses and patients to discuss the MR examination process problems, encourage patients to express their feelings, analyze the underlying causes, and collaboratively devise effective solutions.
5. Evaluating progress: The nurses assess the efficacy of the interventions, conduct an analysis of the deficiencies, investigate the underlying causes, and synthesize their findings at the conclusion of the examination.

### 2.5. Definition of a claustrophobic event

The occurrence of a claustrophobic event is defined as the premature termination of the examination or the administration of intravenous or oral sedation at any time during the examination.^[25]^ When a claustrophobic event occurs, it is categorized according to the severity of the event as mild (non-sedation coping actions), moderate (intravenous or oral sedation), and severe (premature termination). When patients encountered multiple degrees of claustrophobic events during the examination, we only conducted statistical analysis on the most severe events.

### 2.6. Statistical analysis

The database was established by using the method of double entry, and the data were analyzed by applying SPSS (Version 21.0, IBMCorp, Armonk) and Origin (Version 2021, OriginLab, Northampton ). Shapiro–Wilk test was employed to assess the normality of the data. Data conforming to a normal distribution were subjected to an independent sample *t* test and presented as mean ± SD ($\bar{x}\pm s$). For data with a non-normal distribution, the Mann–Whitney *U* test was used and expressed as median (25%, 75%). Qualitative information was expressed as number of cases and percentage (%), and comparisons were made using the *χ*^2^ test. A *P* value equal to or smaller than 0.05 was considered significant.

## 3. Results

During this study, we performed examinations of 223 patients on a 3.0-T MR scanner (MAGNETOM Trio 3.0T, SIEMENS, Erlangen, Germany). All patients in both the intervention and control groups signed written informed consent, and the CLQ questionnaire was administered to all of them both before and after the MR examination. No patients were included in both the control and intervention groups. All patients eventually passed the examination. At the end of the examination, we counted all the patient’s claustrophobic events that occurred during the examination and the corresponding response measures, the average time of examination completion, the 1-time pass rate, the quality of the examination images, the patients’ self-reported examination satisfaction, and the scores of the HADS questionnaire and the CD-RISC questionnaire. We discovered that the mean CLQ values for both groups in this study exceeded the established cutoff value of 0.33, indicating that the sample population utilized in highly representative.^[26]^ Figure 1 illustrates the flowchart for study inclusion. The descriptive statistics for both the intervention and control groups are presented in Table 2, and the detailed anatomical region information can be found in Table 3. Table 4 shows the average completion time and 1-time pass rate of the 2 groups, while Table 5 provides information on the image quality and the patient’s satisfaction with the examination. Table 6 reflects the CLQ, HADS, and CD-RISC scores before and after the examination in both groups.

**Table 2:** Demographics and predictors of the intervention group (SFBT & MBSR) compared to control group (routine psychological care).

	Intervention group (n = 77)		Control group (n = 146)	
Sex				
Male	55%	(42/77)	48%	(70/146)
Female	45%	(35/77)	52%	(76/146)
Age	50.1 ± 11.9	[21–77]	48.7 ± 12.9	[22–75]
Claustrophobic events (total)	18%	(14/77)	46%	(67/146)
Premature termination	4%	(3/77)	13%	(19/146)
Sedation	3%	(2/77)	10%	(15/146)
Non-sedation coping actions	12%	(9/77)	23%	(33/146)

Data are given in percentage (nominator/denominator) or mean ± SD and [min–max].

MBSR = mindfulness-based stress reduction, SFBT = solution-focused brief therapy.

**Table 3:** Number of exams from different anatomical regions and corresponding claustrophobic event rate in the intervention group (SFBT & MBSR) and control group (routine psychological care).

Examination	Intervention group (n = 77)		Control group (n = 146)	
	Examinations	Events	Examinations	Events
Combinations	6 (8%)	0	23 (16%)	5 (22%)
Brain/head/neck	34 (44%)	9 (26%)	44 (30%)	28 (64%)
Thorax	1 (1%)	0	6 (4%)	1 (17%)
Abdomen/pelvis	32 (42%)	4 (12%)	59 (40%)	19 (32%)
Upper extremities	1 (1%)	0	6 (4%)	2 (33%)
Lower extremities	3 (4%)	1 (33%)	8 (5%)	2 (25%)

MBSR = mindfulness-based stress reduction, SFBT = solution-focused brief therapy.

**Table 4:** Inspection completion time and 1-time pass rate for the intervention group (SFBT & MBSR) and control group (routine psychological care).

Groups	All number	Completion time	One-time pass rate	
			Number	Rate
Intervention group	77	17.28 ± 3.36	63	82%
Control group	146	19.75 ± 4.23	89	61%
*t* value		5.837	14.793	
*P* value		<.05	<.05	

Data are given in mean ± SD.

MBSR = mindfulness-based stress reduction, SFBT = solution-focused brief therapy.

**Table 5:** Comparison of image quality and patient examination satisfaction for the intervention group (SFBT & MBSR) and control group (routine psychological care).

	Image quality		Examination satisfaction	
	Intervention group	Control group	Intervention group	Control group
All number	77	146	77	146
Very satisfied	59	66	70	97
Satisfied	14	28	3	32
Fair	1	28	3	8
Unsatisfied	2	13	1	4
Very unsatisfied	1	11	0	5
*t* value	4.743		8.907	
*P* value	<.05		<.05	

MBSR = mindfulness-based stress reduction, SFBT = solution-focused brief therapy.

**Table 6:** CLQ, HADS and CD-RISC scores for the intervention group (SFBT & MBSR) compared to control group (routine psychological care).

Group	CLQ				HADS				CD-RISC			
	Before	After	*t* value	*P* value	Before	After	*t* value	*P* value	Before	After	*t* value	*P* value
Intervention group	1.93 ± 1.1	1.92 ± 1.1	0.221	>.05	9.03 ± 0.9	6.30 ± 0.8	3.021	<.05	57.73 ± 4.5	66.08 ± 4.7	8.928	<.05
Control group	1.95 ± 1.2	1.95 ± 1.2	0.136	>.05	9.22 ± 1.0	9.08 ± 1.0	0.803	>.05	57.52 ± 4.7	57.78 ± 4.6	0.740	>.05
*t* value	0.209	0.283			1.396	2.753			0.538	7.749		
*P* value	>.05	>.05			>.05	<.05			>.05	<.05		

Data are given in mean ± SD.

CLQ = Claustrophobia questionnaire, HADS = hospital anxiety and depression scale, CD-RISC = Connor-Davidson resilience scale, MBSR = mindfulness-based stress reduction, SFBT = solution-focused brief therapy.

**Figure 2.**
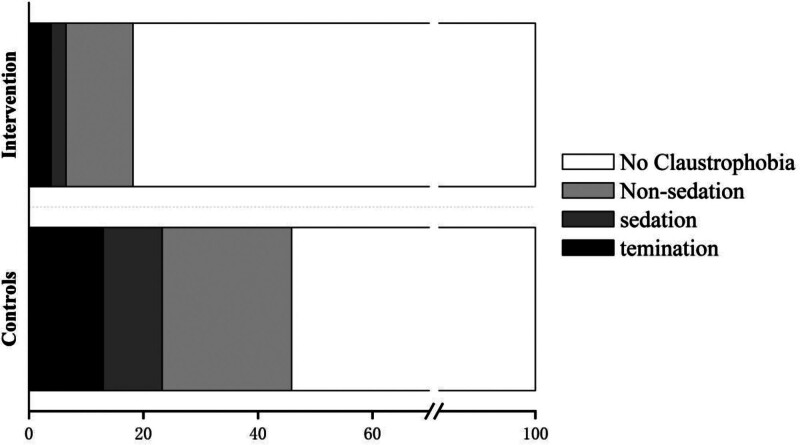
Frequency of claustrophobia (percentage of patients) in intervention and control groups.

The utilization of the intervention that combines SFBT with MBSR can result in a significant reduction in claustrophobic events, decreasing from 46% (67/146) to 18% (14/77). Additionally, there is a decrease in the requirement for sedation from 10% (15/146) to 3% (2/77), as well as a decline in premature terminations from 13% (19/146) to 4% (3/77). Furthermore, non-sedation coping actions are reduced from 23% (33/146) to 12% (9/77, Table 2 and Fig. 2).

By counting the detailed anatomical region scanning information of all patients, we found those who had MR examining of brain/head/neck were more likely to develop claustrophobia events than those who had other regions examined (Table 3). This finding is consistent with the findings of Napp et al.^[25]^ The reason for this may be attributed to the fact that this type of examination typically involves the addition of extracorporeal coils at the appropriate site to enhance image quality. These coils are significantly smaller, which may potentially trigger feelings of claustrophobia.

Compared with the control group, the average completion time of the intervention group was shorter (17.28 ± 3.36 vs 19.75 ± 4.23; *P* < .05), the 1-time pass rate was higher (82% vs 61%; *P* < .05, Table 4), and the quality of the examination images and the patient’s satisfaction with the examination were better (*P* < .05, Table 5), all differences were statistically significant. The use of SFBT & MBSR interventions prior to MR examinations can reduce patients’ anxiety (HADS: from 9.03 ± 0.9 to 6.30 ± 0.8; *P* < .05) and significantly contribute to the building of their psychological resilience (CD-RISC: from 57.73 ± 4.5 to 66.08 ± 4.7; *P* < .05), and the improvement effect is more significant compared with the control group (*P* < .05). Although there was a decrease in HADS scores (9.22 ± 1.0 vs 9.08 ± 1.0) and an increase in CD-RISC scores (57.52 ± 4.7 vs 57.78 ± 4.6) in the control group before and after the examination, the differences were not statistically significant (*P* > .05). The changes in CLQ before and after the intervention were not found to be statistically significant in either the intervention or control group, indicating no significant differences between the 2 groups (*P* > .05). This result is also in alignment with the conclusions drawn from a prior investigation conducted by Napp et al.^[25]^

## 4. Discussion

To the best of our knowledge, we are pioneering the investigation into the impact of combining SFBT with MBSR on claustrophobic events in patients undergoing MR examinations. We conducted comprehensive interviews with all patients who participated in the SFBT & MBSR intervention, and the overwhelming majority reported a high level of acceptability. Six major areas were identified as valuable components of treatment: MBSR, describing the problem, developing well-formed goals, exploring for exceptions, end-of-session feedback, and evaluating progress. This study exclusively enrolled patients with a high susceptibility to claustrophobia (CLQ > 0.33). The participants were divided into 2 groups: the intervention group and the control group. In the intervention group, SFBT & MBSR were administered before the MR examination, while routine psychological care was executed in the control group before the MR examination. The main findings of our study are: In patients with a high risk of claustrophobia, SFBT & MBSR can significantly reduce the chance of claustrophobic events and dramatically reduce the need for sedation and other coping actions during the MR examination. SFBT & MBSR can shorten the MR examination time and increase the 1-time pass rate. SFBT & MBSR can improve the image quality and examination satisfaction of patients with high risk of claustrophobia. SFBT & MBSR can significantly improve psychological resilience and reduce anxiety in patients at high risk of claustrophobia with MR examinations. The efficacy of SFBT & MBSR in improving CLQ among high-risk patients with claustrophobia is limited.

A striking finding from this study is that claustrophobic patients who enter a closed, dark, narrow space may experience psychological reactions such as fear and anxiety. In severe cases, the patient may experience dizziness, panic, dyspnea, agitation, or even asphyxiation, and may not be able to persist in completing the MR examination. Thus, previous claustrophobia is often treated as a contraindication to MR examination. In contrast to sedation and non-sedation coping actions, we provide a new psychological intervention to assist these patients in overcoming the physical and psychological disruption of claustrophobia and to facilitate their successful MR examination. Although no scholars previously used SFBT or MBSR to intervene with claustrophobic patients who were preparing to undergo MR examination, a large number of studies have shown that SFBT, as a low-cost and high-efficacy training mode, can significantly improve patients’ self-efficacy,^[27,28]^ help patients and their families to establish more positive emotional experiences,^[29,30]^ increase patients’ positive health behaviors,^[15,31,32]^ promote patients’ postoperative rehabilitation,^[27,33,34]^ and even change their psychosocial functions.^[35]^ Numerous studies have shown that MBSR plays a significant role in treating negative emotions such as anxiety and depression and recurrence of depression,^[36,37]^ improving cancer-related fatigue and pain in cancer patients,^[36,38,39]^ and improving prognosis by increasing the degree of psychological resilience of patients.^[40,41]^ This study innovatively combined SFBT with MBSR and found that this combined intervention achieved excellent results in patients with claustrophobia who were prepared to undergo magnetic resonance examinations.

Compared with sedation and non-sedation coping actions, this study provides a novel approach for radiologists to ensure that patients can successfully complete their MR examinations. One of the strengths of this study is that this method has a high social acceptance and a positive effect on patients’ subsequent lives. All 77 participants who received the intervention agreed to be interviewed and indicated that they were highly receptive to the treatment and widely recognized its efficacy, with many patients indicating that the treatment would have an additional positive impact on their future daily lives.

Although our findings have some clinical value, it does have several limitations. First of all, the samples in this study were from a single medical institution, with certain regional and specific characteristics, and the sample size was relatively limited, which may affect the external applicability and statistical robustness of the research results. Although we tried our best to improve the representativeness of samples and the scientificity of data in the study design, differences in medical resource allocation, patient background, and clinical practice patterns in different regions may still affect the generalization of research results. In the future, we plan to conduct prospective studies covering different regions, multiple medical centers, and larger sample sizes to evaluate the stability and applicability of the findings of this study in different clinical Settings and patient populations. Second, the study did not adequately address potential confounding factors that could have influenced the results. Factors such as the patient’s pre-study anxiety level, previous MRI experience, and the severity of the condition requiring MRI may influence the intervention’s effectiveness. We plan to include more confounding variables in subsequent studies, build a propensity scoring model, and screen variables based on their actual association with outcomes and data quality to optimize the stability and fit of the model. Third, this study only evaluated the immediate effects of SFBT combined with MBSR intervention, and did not set up follow-up nodes to determine the long-term effects of the intervention on claustrophobia and other outcomes. We plan to conduct 1- and 3-month follow-up studies in the future, combined with qualitative methods, to further evaluate the long-term effects of the intervention on claustrophobia remission and medical adherence. Fourthly, the intervention time in this study was relatively long. Some patients undergoing MR Examination could not complete the intervention process due to the scheduling arrangement, and some patients underwent the examination immediately after the intervention, which may affect their internalization of the intervention content and produce practical effects. Finally, we only compared the difference between SFBT and MBSR combined intervention and conventional psychological care, and did not set up SFBT or MBSR alone intervention groups, so it is difficult to determine the marginal effect of the combined intervention. We plan to set up multiple control groups in follow-up studies to further explore the independent and interactive effects of each intervention.

## 5. Conclusion

The combination of SFBT and MBSR for claustrophobic patients can significantly reduce the risk of claustrophobia, shorten the examination time, improve the image quality, alleviate negative emotions, and increase the patient’s satisfaction with the medical treatment. The SFBT intervention combined with MBSR appears to be a valuable tool for reducing medical sedation and the time medical staff spend on coping strategies, and it holds promise for broader clinical application in the future. We will continue to advance related research to further verify the sustainability and applicability of this intervention.

## Author contributions

**Conceptualization:** Liyuan Fu, Minghui Mao, Hui Xiao.

**Data curation:** Liyuan Fu, Chengkun Hong, Yuhang Zhang, Chujie Chen.

**Formal analysis:** Chengkun Hong, Yuhang Zhang, Chujie Chen, Zhijie Yang, Yamei Lin.

**Writing – original draft:** Liyuan Fu, Minghui Mao.

**Writing – review & editing:** Liyuan Fu, Hui Xiao, Hao Huang.
